# Feasibility of the preparation of cochleate suspensions from naturally derived phosphatidylserines

**DOI:** 10.3389/fmedt.2023.1241368

**Published:** 2023-09-06

**Authors:** Søren Kristensen, Khadeija Hassan, Nadia Skarnager Andersen, Frank Steiniger, Judith Kuntsche

**Affiliations:** ^1^ Department of Physics, Chemistry and Pharmacy, University of Southern Denmark, Odense, Denmark; ^2^ Center for Electron Microscopy, Jena University Hospital, Jena, Germany

**Keywords:** cochleates, phosphatidylserine, drug delivery, lipid formulation, physicochemical characterization, electron microscopy

## Abstract

**Introduction:**

Cochleates are cylindrical particles composed of dehydrated phospholipid bilayers. They are typically prepared by addition of calcium ions to vesicles composed of negatively charged phospholipids such as phosphatidylserines (PS). Due to their high physical and chemical stability, they provide an interesting alternative over other lipid-based drug formulations for example to improve oral bioavailability or to obtain a parenteral sustained-release formulation.

**Methods:**

In the present study, the feasibility to prepare cochleate suspensions from soy lecithin-derived phosphatidylserines (SPS) was investigated and compared to the “gold standard” dioleoyl-phosphatidylserine (DOPS) cochleates. The SPS lipids covered a large range of purities between 53 and >96% and computer-controlled mixing was evaluated for the preparation of the cochleate suspensions. Electron microscopic investigations were combined with small-angle x-ray diffraction (SAXD) and Laurdan generalized polarization (GP) analysis to characterize particle structure and lipid organization.

**Results:**

Despite some differences in particle morphology, cochleate suspensions with similar internal lipid structure as DOPS cochleates could be prepared from SPS with high headgroup purity (≥96%). Suspensions prepared from SPS with lower purity still revealed a remarkably high degree of lipid dehydration and well-organized lamellar structure. However, the particle shape was less defined, and the typical cochleate cylinders could only be detected in suspensions prepared with higher amount of calcium ions. Finally, the study proves the feasibility to prepare suspensions of cochleates or cochleate-like particles directly from a calcium salt of soy-PS by dialysis.

## Graphical Abstract

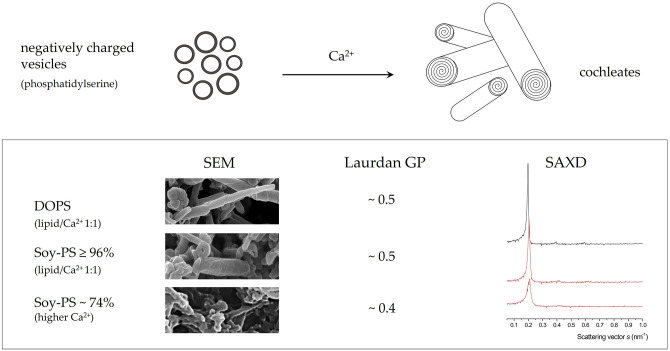

## 1. Introduction

Cochleates, which have first been described by Papahadjopoulos in 1975 (1) are formed by specific binding of multivalent cations such as calcium ions to negatively charged phospholipids such as phosphatidylserine (PS). The binding of calcium ions results in dehydration of the phospholipid headgroup, collapse and fusion of the vesicles followed by formation of lamellar sheets which roll up to form cochleate cylinders (1–3). By addition of EDTA, a calcium chelator, the cochleates lose their structure and large vesicles are obtained again (1). Due to their solid structure, cochleate particles possess a considerably high chemical and physical stability, what makes them interesting for drug formulation (4, 5). The predominant application for cochleate suspensions is oral drug delivery and the prospect of this formulation strategy could clearly be illustrated for amphotericin B-loaded cochleate suspensions (6–11). However, cochleate formulations have generally been suggested for a broad range of drug administration and the interested reader is referred a recent comprehensive review article (5).

In the first studies on cochleate formation, isolated phosphatidylserine from bovine brain with high purity has been used (1, 2), but synthetic phospholipids, especially DOPS, have then mostly been applied to prepare cochleates (6–8, 12) and especially for structural analysis (3, 13, 14). From an industrial perspective of drug development, costs and availability of the excipients are highly relevant and cheaper alternatives such as naturally derived phosphatidylserines are of interest especially for oral drug delivery (15). Accordingly, the use of phosphatidylserines derived from soy lecithin has been described in patent applications (16, 17). In addition, some recent studies used soy-phosphatidylserine to prepare cochleates incorporating lipid-A as an oral vaccine adjuvant system (18) and amphotericin B for oral administration (9), respectively. However, there is still very limited information about the morphology and lipid structure in cochleate suspensions prepared from natural phosphatidylserines in the scientific literature.

**SCHEME 1:**
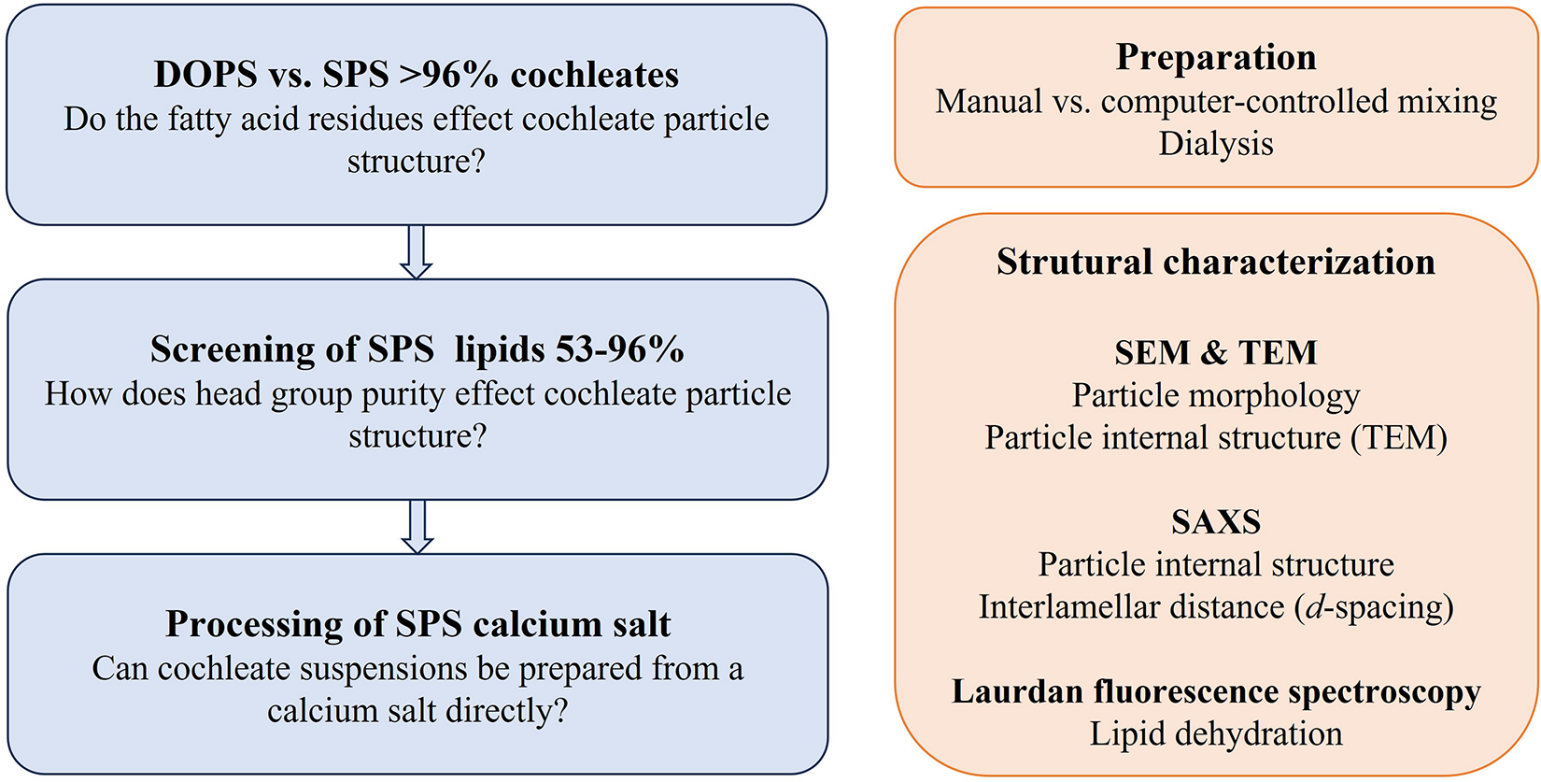
Graphical presentation of the study design.

The aim of the present study was thus to explore particle structure and lipid organization in cochleate suspensions prepared by phosphatidylserines derived from soy lecithin in comparison to the gold standard DOPS (Scheme 1) and applying different experimental methods for comprehensive structural analysis. Phosphatidylserine can be obtained from lecithin by enzymatic headgroup modification (19) or by precipitation of the fraction of negatively charged lipids from raw lecithin followed by purification and eventually recrystallization into the water-soluble sodium salt. In the present work, seven phosphatidylserines derived from soy lecithin differing in headgroup purity and salt form (sodium vs. calcium salt) have been studied (Table 1). In addition, the feasibility of direct processing the calcium salt into a cochleate formulation has been evaluated. This is of special practical and economical interest, as this could eliminate additional processing steps upon PS isolation and purification from lecithin.

**Table 1:** Specifications of the soybean lecithin-derived lipids according to the specifications from the manufacturer (lipoid). The sample codes derived from the batch numbers (numbers in bold).

Code	Batch number	PS (%)	PA (%)	PC (%)	Cation	Description
SPS-10	599,990–21600**10**/001	≥96	4	0.4	Na^+^	Enzymatic modification from purified soybean PC
SPS-13	599,990–21600**13**/001	65	23	2	Na^+^/Ca^2+^	Enzymatic modification from soybean PC
SPS-14	599,990–21600**14**/001	55	20	5	Na^+^/Ca^2+^	SPS13 mixed with soybean lecithin
SPS-16	599,990–21700**16**/001	73	12	N/A	Na^+^	SPS-15 converted to Na^+^ salt
SPS-15	599,990–21700**15**/001	65	12	N/A	Ca^2+^	Calcium salt of PS from soybean lecithin
SPS-19	599,990–21800**19**/914	74	N/A	N/A	Ca^2+^	Calcium salt of PS from soybean lecithin
SPS-34	538,500–21600**34**/911	53	N/A	N/A	Ca^2+^	Calcium salt of PS from soybean lecithin

PS, phosphatidylserine; PA, phosphatidic acid; PC, phosphatidylcholine.

For comprehensive characterization of lipid organization in the cochleate cylinders, electron microscopy was combined with small-angle x-ray diffraction (SAXD) and Laurdan generalized polarization (GP) analysis. Whereas scanning electron microscopy (SEM) provides information about the particle shape and surface, the particle inner structure and lipid organization can be visualized by cryo-transmission electron microscopy (cryo-TEM) as well as TEM of thin cross sections of the sample (3, 20). Information about the lipid organization can also be determined by SAXD. The highly ordered, dehydrated lamellar arrangement of the lipids in the cochleate structure results in a very sharp 1st order reflection in the small-angle range indicating the thickness of the lipid lamellae [5.1 and 5.2 nm for DSPS and DOPS cochleates, respectively (14, 21)]. Information about membrane rigidity and lipid dehydration can be obtained by analyzing the Laurdan GP (22). The energy of Laurdan's emission depends on the polarity of the environment of the headgroup of the Laurdan molecule. In a polar environment, the fluorophore loses some energy due to dipole-dipole interactions and emission is shifted to higher wavelengths. This effect can be used to investigate membrane rigidity and structure (23, 24). In a fluid membrane, some Laurdan molecules will be deep inside the membrane (non-polar environment, emission wavelength around 430 nm), whereas others are close to the hydrated headgroups of the phospholipids and thus in contact with water molecules (emission wavelength around 490 nm). By setting the intensities at both wavelength in relation to each other, the general polarization (GP) can be calculated on a scale from −1 (only polar) to 1 (only non-polar).

**SCHEME 2:**
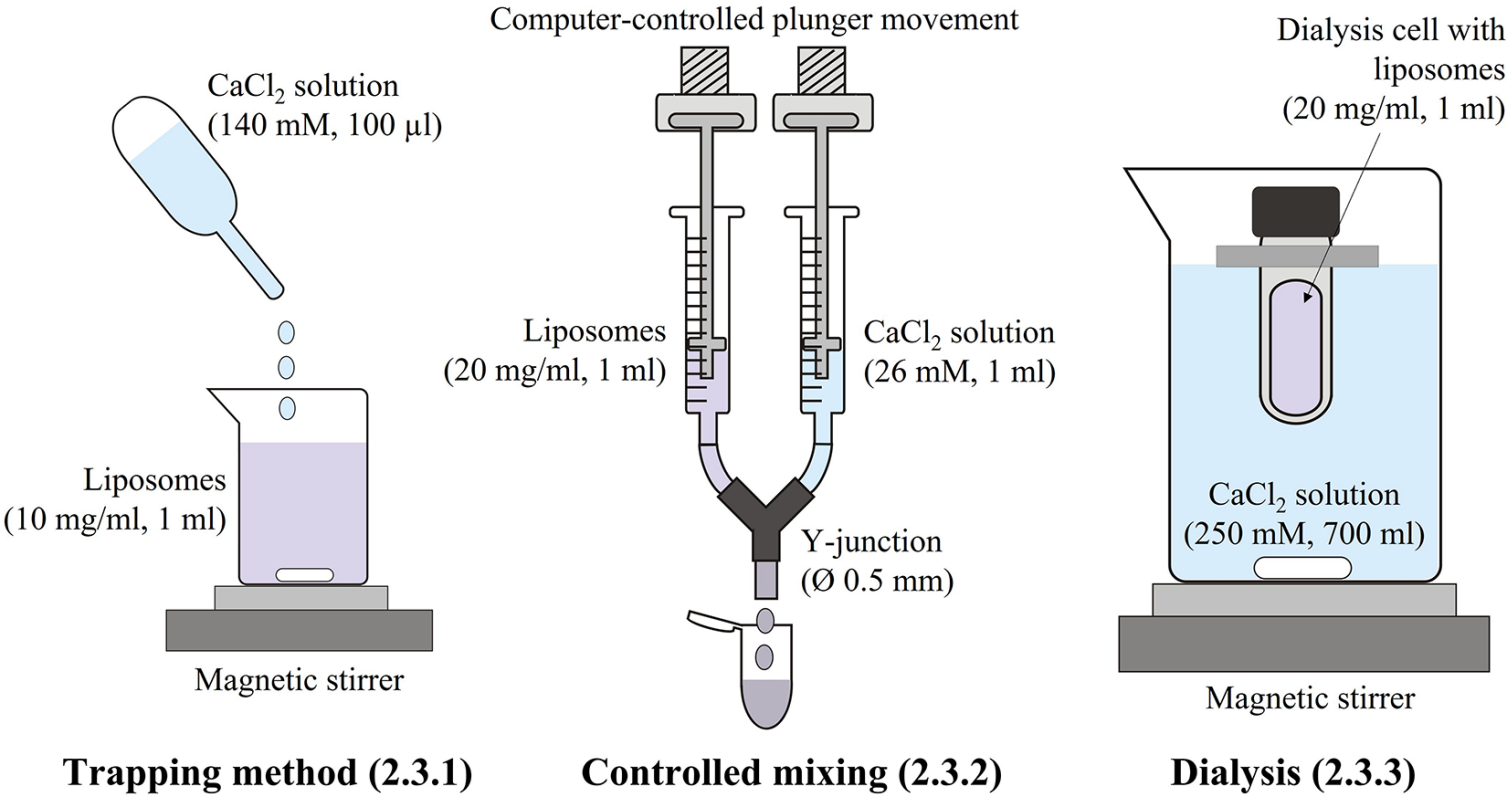
Schematic presentation of the methods used for the preparation of the cochleate suspensions.

Different methods for the preparation of cochleate suspensions have been described in the literature (3, 12, 13, 25) where the simple addition of calcium chloride solution to the PS-vesicle suspension (“trapping method”) was selected as a starting point in this study. However, as standardization of manual addition of the calcium chloride solution is difficult to achieve, preparation of cochleate suspensions by using a computer-controlled mixing device and by dialysis was evaluated (Scheme 2).

## 2. Materials and methods

### 2.1. Materials

Dioleoyl phosphatidylserine (DOPS, ≥99%) was obtained from Avanti Polar Lipids Inc. (U.S.) and soybean phosphatidylserines (SPS) of varying purities (Table 1) were provided by the Lipoid GmbH (Germany). TRIZMA preset-crystals pH 7.4, calcium chloride dihydrate (Ph.Eur.), sodium azide (≥99.5%), EDTA tetrasodium dihydrate (>99%) and chloroform (≥99.0%, Ph.Eur., stabilized with ∼1% ethanol) were obtained from Sigma, sodium chloride (Ph.Eur.) from VWR and Laurdan (6-dodecanoyl-2-dimethylaminonaphtalene) from Molecular Probes (Thermo-Fisher Scientific). Purified water was obtained from a Milli-Q Advantage A10 system (Millipore).

### 2.2. Preparation of liposomes

Liposomes (20 mg/ml lipid) were prepared either by the lipid-film method or by directly dispersing the lipid in buffer under mechanical agitation (Heidolph Multi-reax set to 500 rpm; Heidolph Instr., Germany) overnight. If not stated otherwise, 10 mM Tris buffer pH 7.4 preserved with 0.02% (w/v) sodium azide (Tris buffer) was used. To facilitate dispersion of the SPS lipids containing calcium ions, an adequate amount of EDTA was added to the buffer (Table 2). The crude liposome dispersions were submitted to bath sonication (35 kHz, Bandelin Sonorex Digitech, 2–6 cycles à 15 min, Bandelin electronic GmbH & Co. KG, Germany) to obtain suspensions of small unilamellar vesicles. The liposome suspensions were stored at 4–8°C until use. Specifications of the liposomes used in this study are provided in Table 2.

**Table 2:** Specifications of the liposomes used for cochleate preparation.

Lipid	EDTA (mM)	DLS		Method for cochleate preparation
		Diameter (nm)	PdI	
DOPS	0	65 ± 3	0.216 ± 0.018	TM, CM
SPS-10	0	103 ± 4	>0.5	TM, CM
SPS-10	10	37 ± 1	0.238 ± 0.001	CM
SPS-13	10	70 ± 1	0.238 ± 0.001	CM
SPS-14	10	71 ± 1	0.230 ± 0.005	CM
SPS-15	30	36 ± 1	0.222 ± 0.004	CM
SPS-16	10	36 ± 1	0.224 ± 0.007	CM
SPS-19	25–30	30 ± 1	0.223 ± 0.009	CM, D
SPS-34	30	64 ± 1	0.234 ± 0.004	CM

PdI, polydispersity index; TM, tapping method; CM, controlled mixing (80 µl/s); D, dialysis.

### 2.3. Cochleate preparation

The different methods used for cochleate preparation in this study are schematically presented in Scheme 2.

#### 2.3.1. Trapping method

100 µl 140 mM calcium chloride solution in Tris buffer was added dropwise to 1 ml diluted liposome suspension (10 mg/ml lipid) under magnetic stirring (250 rpm) to reach a lipid/calcium ion molar ratio of about 1:1.

#### 2.3.2. Controlled mixing

Equal volumes of liposome suspension (20 mg/ml) and calcium chloride solution in Tris buffer were mixed at a controlled mixing speed of 80 µl/s. The self-constructed device consisted of two disposable 1-ml syringes with the syringe plungers connected to each a motor and the syringe outlets to a Y-junction (inner diameter 0.5 mm). If not stated otherwise, the calcium chloride concentration was adjusted to reach a lipid/calcium ion molar ratio of 1:1. If necessary, the pH was adjusted to 7.4.

#### 2.3.3. Dialysis

1 ml liposome suspension (20 mg/ml lipid) was filled in a dialysis cell (Pur-A-Lyzer, MWCO 12.5 kDa, Sigma Aldrich) and placed in a 1,000 ml beaker containing 700 ml 250 mM calcium chloride solution in Tris buffer. Dialysis was carried out under magnetic stirring and light protection for 24 h with a buffer change after 2 and 4 h. Samples were withdrawn at predetermined time points from the dialysis cell to monitor the formation of cochleates.

### 2.4. Dynamic light scattering (DLS)

Vesicle size of the liposome suspensions was determined by DLS (DelsaMax Pro, Beckman Coulter Life Science, U.S.). The diluted (1:1,000 in Tris buffer) liposomes were measured 6 times over 10 s at 20°C in backscattering mode (163.5°). The hydrodynamic diameter (z-average) and polydispersity index (PdI) were calculated by the instrument's cumulant analysis (DelsaMax version 1.0.1.6. Beckman Coulter). Results given as average and standard deviation of the six acquisitions.

### 2.5. Scanning electron microscopy (SEM)

#### 2.5.1. Conventional SEM

The diluted (1:10 or 1:5) sample was placed on a polycarbonate filter (0.5 × 0.5 cm, 0.4 µm pore size, Whatman Nuclepore Track-etched membrane, Sigma) and air-dried on top of a filter paper. The dried sample was then placed on an aluminum SEM specimen stub and coated with 15-nm gold layer (JFC-1100, Jeol Ltd., Japan). The samples were examined in a LEO-435VP SEM (accelerating voltage 10 kV) or a Quanta 2,000 SEM (FEI, U.S., acceleration voltage 15–20 kV) and images were acquired with Everhart-Thornley detectors.

#### 2.5.2. High-resolution SEM

The diluted (1:10 or 1:5) sample was sonicated for 4 min at room temperature (Elmasonic P, Elma Schmiedhauer GmbH, Germany, 37 kHz, 50% power) and 5 µl sample was then placed on a copper grid (Quantifoil R, 1.2/1.3, Quantifoil Micro Tools GmbH, Germany). The sample was rinsed with a small amount of Tris buffer before being air-dried on top of a filter paper. The copper grid was then placed on an aluminum SEM specimen stub and coated with 7-nm platinum layer (CCU-010, Safematic GmbH, Switzerland). The samples were examined in a LEO 1,530 Gemini SEM (Carl Zeiss GmbH Jena, Germany) operated at 4 kV acceleration voltage. Images were acquired by the InLens detector.

#### 2.5.3. Cryo-SEM

One droplet of the sample was placed on a gold sample carrier BU012 129-T (BAL-TEC AG, Lichtenstein), and rapidly frozen by plunge-freezing in liquid nitrogen-cooled propane/ethane (50:50). The samples were then transferred into a VCT 100 cryo-transfer system (BAL-TEC AG, Lichtenstein), which was continuously cooled with liquid nitrogen. After connecting the VCT 100 system to a MED 020 high-vacuum coating system (BAL-TEC AG, Lichtenstein), samples were fractured and deep-etched at −95°C for 5 min and finally sputter-coated with 2–3 nm gold. Using the VCT 100 cryo-transfer system, samples were finally transferred under vacuum and cryo conditions into a scanning electron microscope Leo 1,530 Gemini (Carl Zeiss GmbH Jena, Germany) onto a liquid nitrogen cooled (at −140°C) sample holder. Images were recorded digitally with an InLens SE detector (Carl Zeiss GmbH Jena, Germany) at 4 kV acceleration voltage.

### 2.6. Transmission electron microscopy (TEM)

#### 2.6.1. Conventional TEM

A small droplet of non-diluted formulation was placed on a holy carbon grid (Quantifoil R2/1, Quantifoil Micro Tools GmbH, Germany) placed on filter paper and air dried for five minutes. The samples were viewed in a transmission electron microscope (CM120, Philipps, Netherlands) at 120 kV. Images were recorded with a 2k CMOS Camera (F216, TVIPS GmbH, Germany).

#### 2.6.2. Cryo-TEM

A drop of diluted (1:1 in Tris buffer) sample was placed on a holey copper grid (Quantifoil R 1.2/1.3, 400 mesh) and rapidly frozen in liquid ethane (about −180°C). A cryo-transfer unit (Gatan 626, Gatan Inc., U.S.) was used to transfer the frozen specimen into the pre-cooled cryo-transmission electron microscope (CM 120, Philipps, Netherlands). The specimen was viewed under low dose conditions (120 kV), and images were recorded with a CCD camera (FastScan F114, TVIPS GmbH, Germany).

#### 2.6.3. Resin-embedded cross sections

Samples were prepared as described previously (3). Briefly, the sample was pelleted by centrifugation and stained in 100 mM cacodylate buffer pH 7.4 with 1% osmium tetroxide for 2 h prior dehydration in 50% ethanol. The dehydrated sample was embedded in epoxy resin Araldite CY 212 (Agar Scientic Ltd., UK). After polymerization, the block was cut into thin sections (70–100 nm) using an Ultracut E ultramicrotome (Reichert-Jung, Germany) at room temperature. Sections were placed on copper grids and examined in the TEM at 120 kV (CM 120, Philipps, Netherlands). Repeat distances of the bilayer structure were determined directly from the TEM images of by analyzing 100 × 100 nm section of the image by Fourier Transform (FFT) in ImageJ 1.52p.

### 2.7. Small-angle x-ray diffraction (SAXD)

Small-angle x-ray diffraction patterns were recorded at room temperature with a SAXSess mc2 instrument (Anton Paar GmbH, Austria, x-ray wavelength: 0.154 nm, CCD-SCX 4,300 detector) using a flow-through capillary. Each sample was measured 50 times over 30 s (1,500 s in total) and desmearing of the raw data was performed with SAXSquant software. The lamellar repeat distance (*d*-spacing) was calculated from the first order reflection according to Bragg's equation with *λ* the wavelength of the x-rays (0.154 nm, Cu Kα) and *θ* the scattering angle:

(1) $$d=\frac{\lambda }{2\;\sin\theta }$$

In the figures, the scattering intensities are plotted against the scattering vector *s* with *s* = 2sin*θ*/*λ* = 1/*d*.

### 2.8. Laurdan fluorescence spectroscopy

10 µl 0.1 mM Laurdan solution in anhydrous ethanol was mixed with 500 µl diluted sample (0.5 mg/ml lipid) and equilibrated under light protection on a shaker (IKA Vibrax IKA GmbH & Co. KG, Germany, 250 rpm). Fluorescence emission spectra were recorded with a Cary Eclipse instrument (Varian, Agilent, U.S.) at room temperature from 380 nm to 600 nm and an excitation wavelength of 370 nm. Generalized polarization (GP) values were calculated taking the fluorescence intensities at 430 nm and 490 nm into account:

(2) $$GP=\frac{I_{430}-I_{490}}{I_{430}+I_{490}}$$

## 3. Results

### 3.1. Comparison of DOPS and SPS ≥ 96% (SPS-10)

To evaluate the general feasibility to prepare cochleate suspensions from naturally derived lipids with similar well-organized lipid organization as found in DOPS cochleates, the soy-PS with highest head-group purity (≥96%; SPS-10) was applied. Cochleate suspensions were prepared by the standard trapping method (10 mg/ml lipid, lipid/calcium ion molar ratio of 1:1).

**Figure 1:**
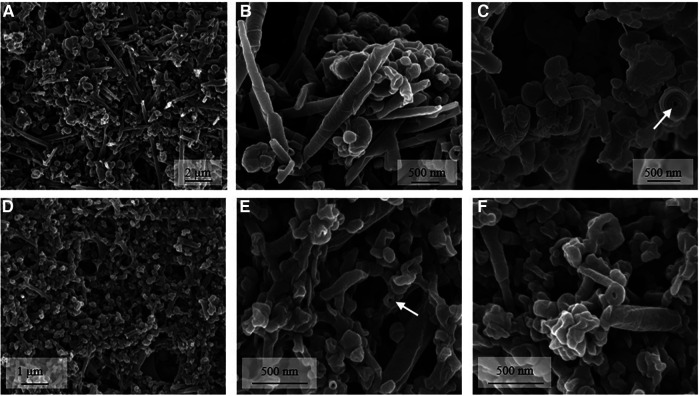
Representative high-resolution SEM (**A,B,D–F**) and cryo SEM (**C**) images of DOPS (**A–C**) and SPS-10 (**D–F**) cochleate suspensions. Cochleate suspensions were prepared by the trapping method at a lipid/calcium ion molar ratio of 1:1. Selected cochleate cylinders with an inner water channel are marked with an arrow. Additional SEM images are presented in the supplementary material (Supplementary Figures S1 and S2).

**Figure 2:**
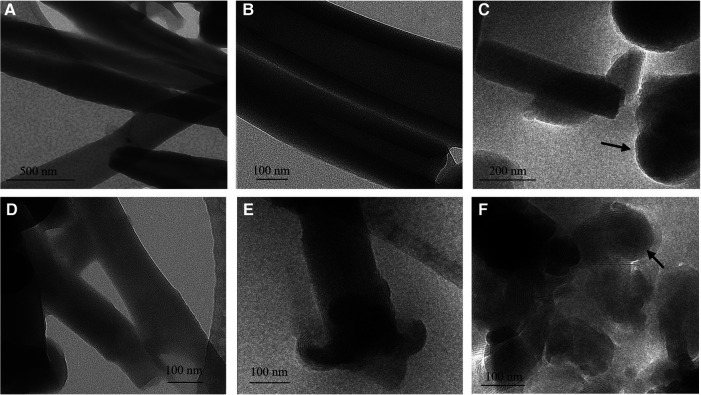
Representative TEM (**A,B,D**) and cryo-TEM (**C,E,F**) images of DOPS (**A–C**) and SPS-10 (**D–F**) cochleate suspensions. Cochleate suspensions were prepared by the trapping method at a lipid/calcium ion molar ratio of 1:1. A spherical particle with well-organized lamellar structure i marked with an arrow in c and f.

**Figure 3:**
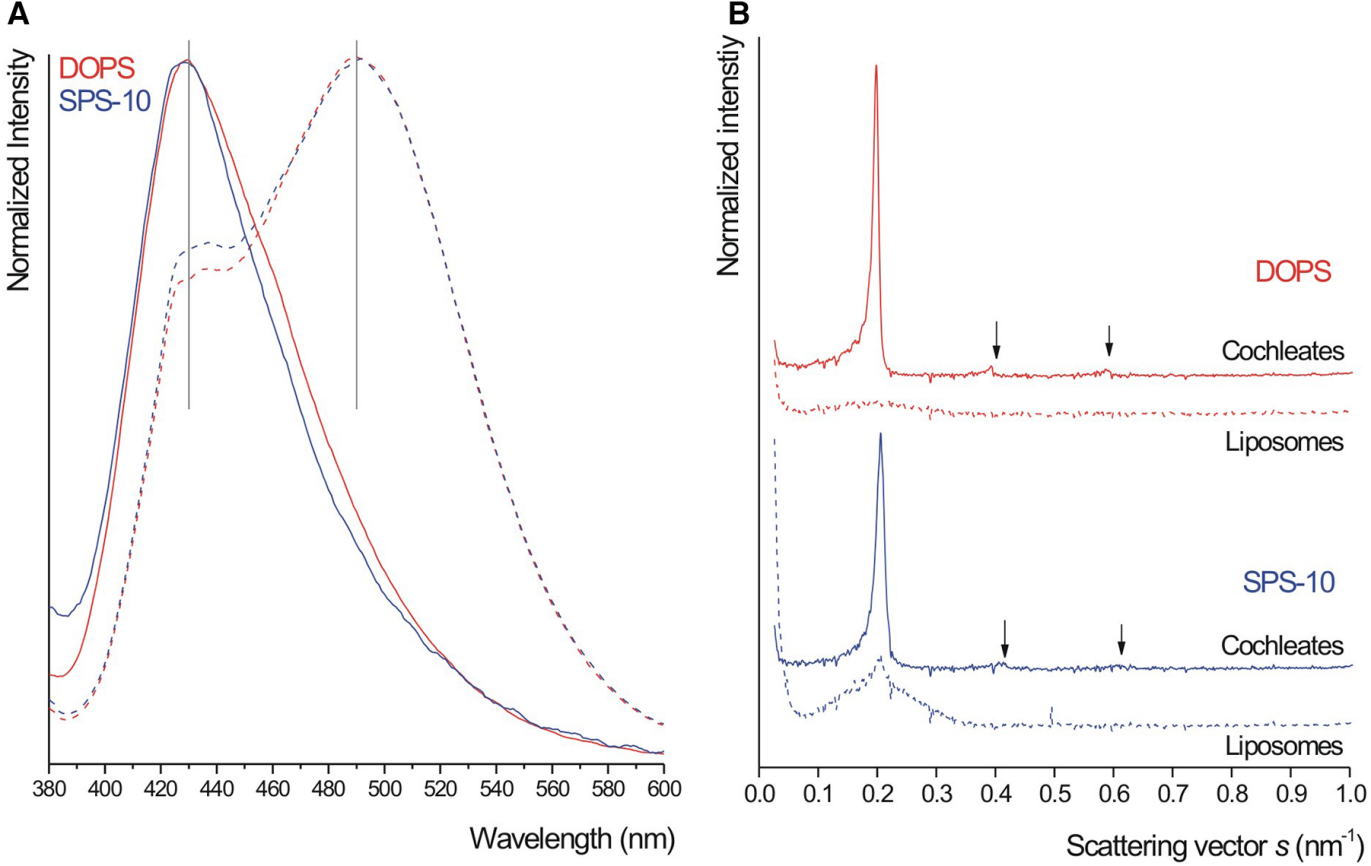
Laurdan emission spectra (**A**) and small-angle x-ray diffraction (SAXD) patterns (**B**) from DOPS (red) and SPS-10 (blue) liposome (dashed lines) and cochleate (closed lines) suspensions. Note the presence of second and third order reflections in (**B**) Cochleate suspensions were prepared by the trapping method at a lipid/calcium ion molar ratio of 1:1.

Addition of calcium ions to the liposome suspensions resulted in immediate flocculation for both lipids. In the DOPS suspension, the typical cochleate structures with high aspect ratio were observed in SEM in addition to more spherical particles (Figures 1A–C). In good agreement with literature (3, 13), some of the cochleate cylinders had an inner water channel (marked with arrows in Figure 1C). In the SPS-10 suspensions, the particles were considerably smaller, but the elongated particles indicate the formation of cochleate cylinders (Figures 1D–F). As in the DOPS suspension, the cochleate cylinders were coexisting with more spherical particles. Due to the solid nature of the particles, the formulations could directly be visualized in TEM (e.g., direct analysis of the dried sample) where they had a rather similar appearance as observed in cryo-TEM (Figure 2). The highly organized lamellar internal structure of the cochleate particles could be visualized for both DOPS (Figures 2A–C) and SPS-10 (Figures 2D–F) cochleates. The lamellar repeat distances of the lipid lamellae were 5.1 ± 0.1 nm (*n* = 11) and 4.8 ± 0.1 nm (*n* = 13) for DOPS and SPS-10 cochleates, respectively. Importantly, the tightly packed lamellar structure could also be visualized in the more spherically shaped particles (marked in Figures 2C,F). To get more quantitative information about the lipid dehydration, Laurdan GP analysis was carried out. As expected, the Laurdan emission spectra of DOPS and SPS-10 liposomes were bimodal (Figure 3A, dotted lines) resulting in slightly negative GP values between −0.1 and −0.2, as typically for membranes in the fluid state. Binding of calcium ions results in dehydration of the phospholipid headgroups and formation of an anhydrous multilamellar structure. Accordingly, a distinct change in the Laurdan emission spectra with an emission maximum around 430 nm was observed (Figure 3A, closed lines). Similar GP values around 0.5 were measured for both DOPS and SPS-10 cochleates indicating a similar degree of dehydration of the lipid bilayers. SAXD results confirmed the well-organized lamellar structure of the lipid particles (Figure 3B, closed lines) by the presence of a very sharp 1st order reflection. Even the much weaker reflections of 2nd and 3rd order (marked with arrows in Figure 3B) could be detected. Based on the position of the reflections, the thickness of the lipid lamella (d-spacing) was determined to be of 5.1 and 4.9 nm for the DOPS and SPS-10 cochleates, respectively. In contrast, only weak and broad reflections were measured for the corresponding liposome suspensions (Figure 3B, dotted lines). Interestingly, the reflection was more distinct for SPS-10 than DOPS liposomes which may be explained by the presence of residual calcium ions in the starting lipid. As the calcium salt of PS cannot be dispersed in an aqueous medium, some crystalline lipid material will be present in the liposome suspension. This is likely also the reason for the larger diameter and PdI in DLS measurements of these liposomes (Table 2). This explanation is supported by the observation that addition of small amounts of EDTA upon liposome preparation resulted in a distinctly smaller size and similar PdI values as measured for the other vesicle suspensions (Table 2).

Despite the differences in particle shape, the results clearly indicate that cochleates with similar structural lipid organization as in DOPS cochleates can be prepared from naturally derived phosphatidylserines with high headgroup purity. Similarly as DOPS cochleates, the SPS-10 cochleate suspensions were physically stable (particle structure and redispersibility) for at least half a year.

DOPS and SPS-10 cochleate suspension were also prepared by controlled mixing of equal volumes of liposomes and calcium chloride solution at a mixing speed of 80 µl/s where suspensions with similar properties were obtained (Supplementary Figure S3). All further samples were thus prepared by controlled mixing at 80 µl/s if not stated otherwise.

### 3.2. Screening of naturally derived phosphatidylserines with different purities

In the next step, a range of naturally derived phosphatidylserines of varying purities (PS between 53 and 96%) and salt forms (sodium or calcium salt or a mixture of both, Table 1) were screened for their ability to form cochleates. To facilitate dispersion of the lipid, EDTA was added in an adequate amount (Table 2) and the concentration of calcium chloride solution used for cochleate preparation was adjusted accordingly to reach lipid/calcium ion molar ratios of about 1:1 or 1:2 for the sodium (SPS-10, SPS-13, SPS-14) and calcium salts (SPS-15, SPS-19, SPS-34), respectively (Table 2).

**Figure 4:**
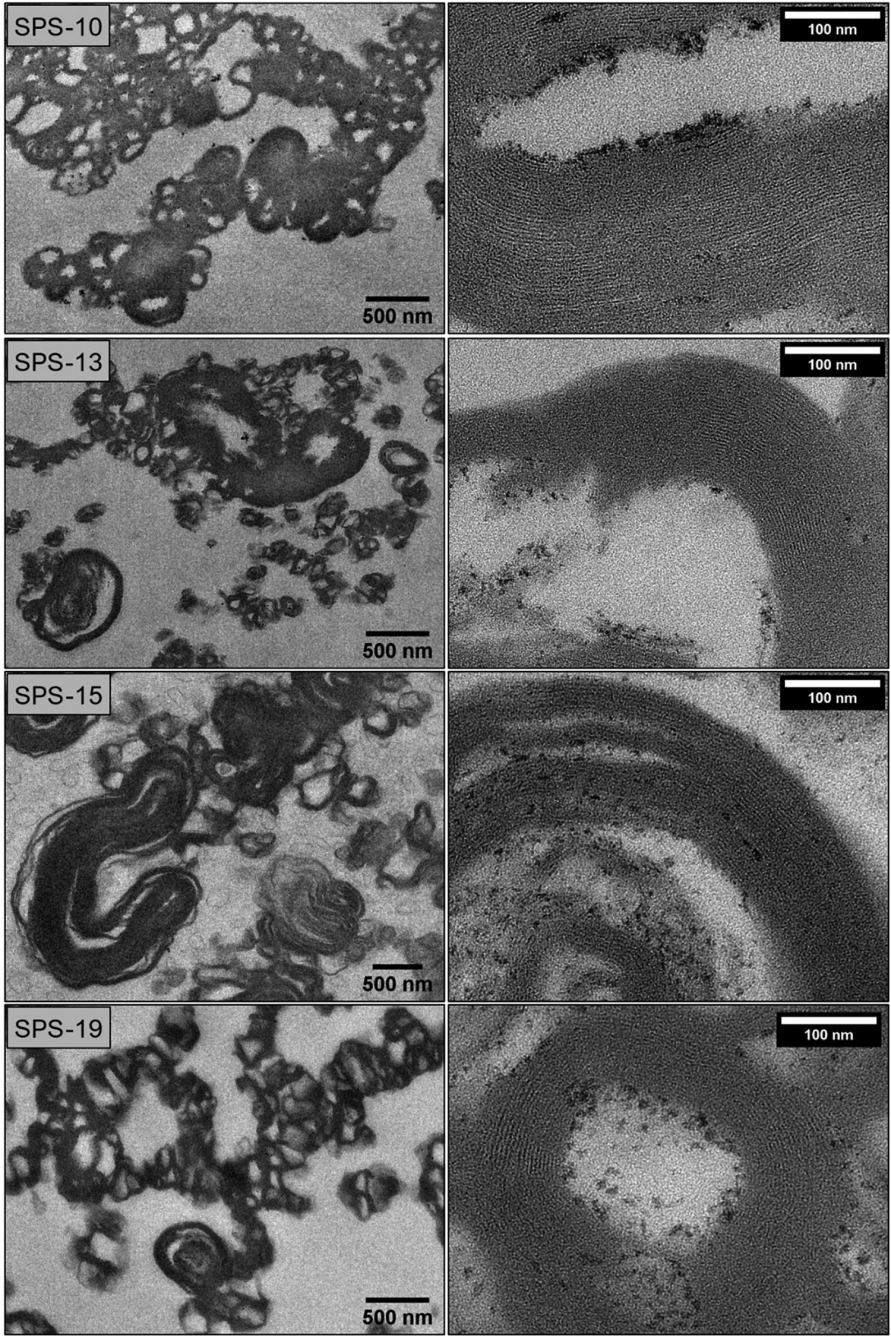
TEM images of resin-embedded samples of cochleate suspensions prepared from SPS of varying purities (SPS-10, SPS-13, SPS-15, SPS-19, controlled mixing, 80 µl/s). The buffer for liposome preparation contained 100 mM sodium chloride and EDTA (Table 2). The calcium chloride concentration was adjusted according to the added EDTA to reach a lipid/calcium ion molar ratio of about 1:1 (sodium salts) or 1:2 (calcium salts). See Supplementary Figure S5 for details on determination of the lamellar thickness.

**Figure 5:**
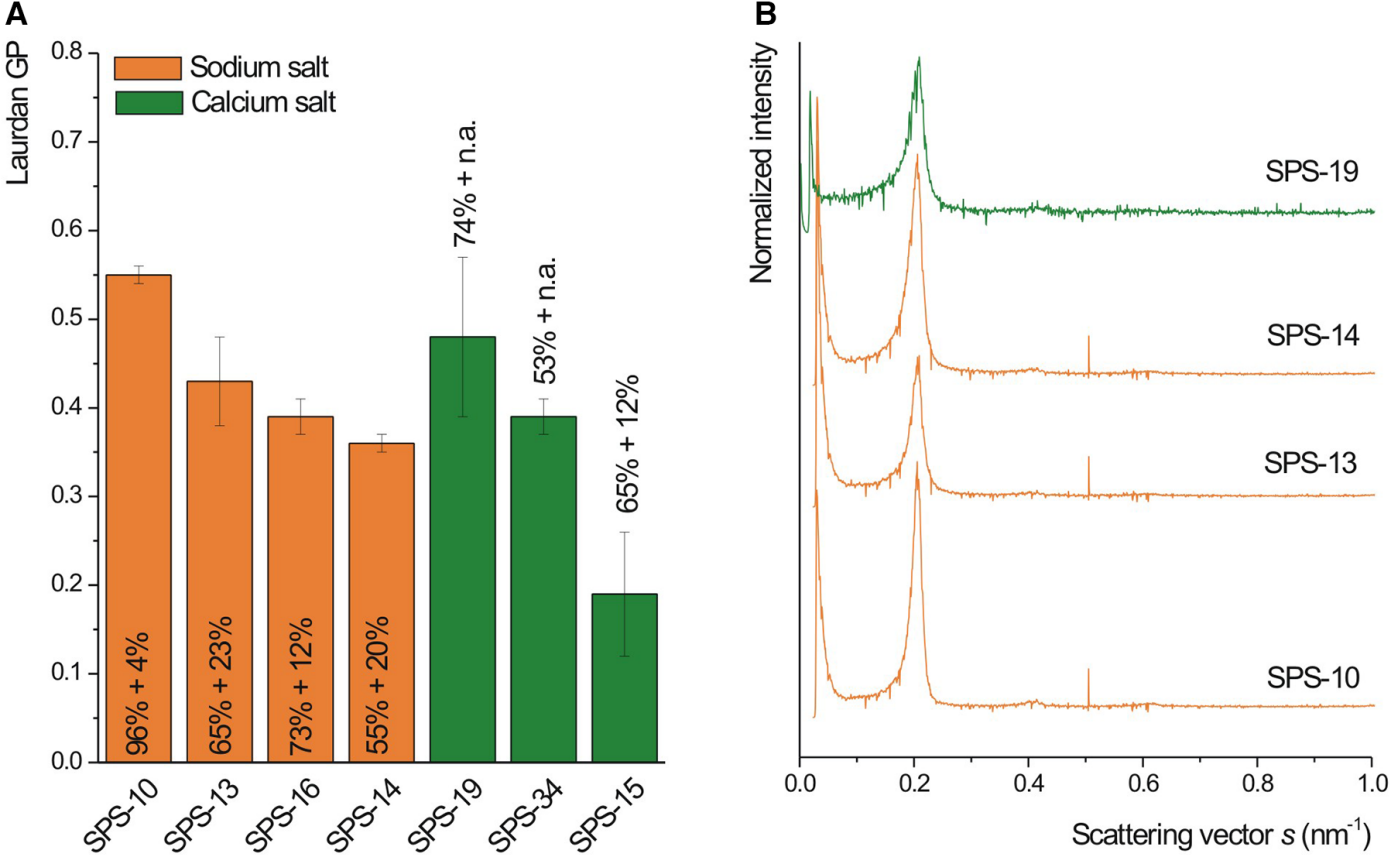
Laurdan GP (**A**, *n* = 3) and SAXD pattern (**B**) of cochleate suspensions prepared from SPS of varying purities (table 1, controlled mixing, 80 µl/s). The mass percent of negatively charged lipids (PS + PA) is indicated in the figure. The buffer for liposome preparation contained 100 mM sodium chloride and EDTA (Table 2). The calcium chloride concentration was adjusted according to the added EDTA to reach a lipid/calcium ion molar ratio of about 1:1 (sodium salts) or 1:2 (calcium salts).

All samples showed immediate flocculation upon mixing with calcium ions, but the characteristic cochleate structures (e.g., cochleate cylinders) could not be seen in SEM (Supplementary Figure S4). To get more information about the inner structure of the particles, selected suspensions (SPS-10, SPS-13, SPS-15 and SPS-19) were stained with osmium tetroxide, embedded in epoxy resin and thin sections were then viewed in the TEM (Figure 4). The overall appearance (images on the left) was similar as observed for DOPS cochleates in a previous study (3). Well-organized lamellar structures could clearly be visualized at higher magnification allowing an estimation of the lamellar repeat distance (see also Supplementary Figure S5). The average lamellar repeat distances were between 4.2 and 4.4 nm (*n* = 4), e.g., somewhat smaller than those determined by TEM original samples and SAXD (4.9 nm). In good agreement with this results, considerably high Laurdan GP values were determined (Figure 5A) with a trend of decreasing GP with decreasing amount of negatively charged lipids. This trend was very clear for the suspensions prepared from the sodium salts (100%, 88%, 85% and 75% of total negatively charged lipids for SPS-10, SPS-13, SPS-16 and SPS-14, Figure 5A). High GP values were also obtained for the SPS-19 and SPS-34 suspensions, which, however, contained a higher amount of calcium ions (lipid/calcium ion molar ratio about 1:2). Moreover, these lipids likely will also contain other (not specified) negatively charged lipids, which may contribute to the high degree of lipid dehydration and organization. In all suspensions, the characteristic SAXD reflection was detected, and the lamellar repeat distance (d = 4.9 nm) was similar for all suspensions. The reflections were, however, broader and less intensive as shown for selected formulations in Figure 5B.

### 3.3. Direct preparation of cochleate suspensions from calcium-SPS

From a practical and economic point of view, direct processing of the water-insoluble calcium salt of PS to prepare the cochleate suspensions is of interest and has been evaluated in this study. Cochleate suspensions were prepared from the most promising SPS calcium salt from the screening experiments (SPS-19). To disperse the water-insoluble calcium PS in the aqueous buffer for liposome preparation, EDTA was added to the buffer (Table 2). Cochleate formulations with different lipid/calcium ion molar ratios (1:1, 1:2, 1:5 and 1:10) were then prepared to determine the optimal composition with respect to homogeneity (aggregate size) and degree of dehydration (high GP values). In addition, a suspension was also prepared by dialyzing liposomes directly against a calcium chloride solution to remove the EDTA, which initially was added for liposome preparation. All cochleate suspensions were prepared in triplicate.

**Figure 6:**
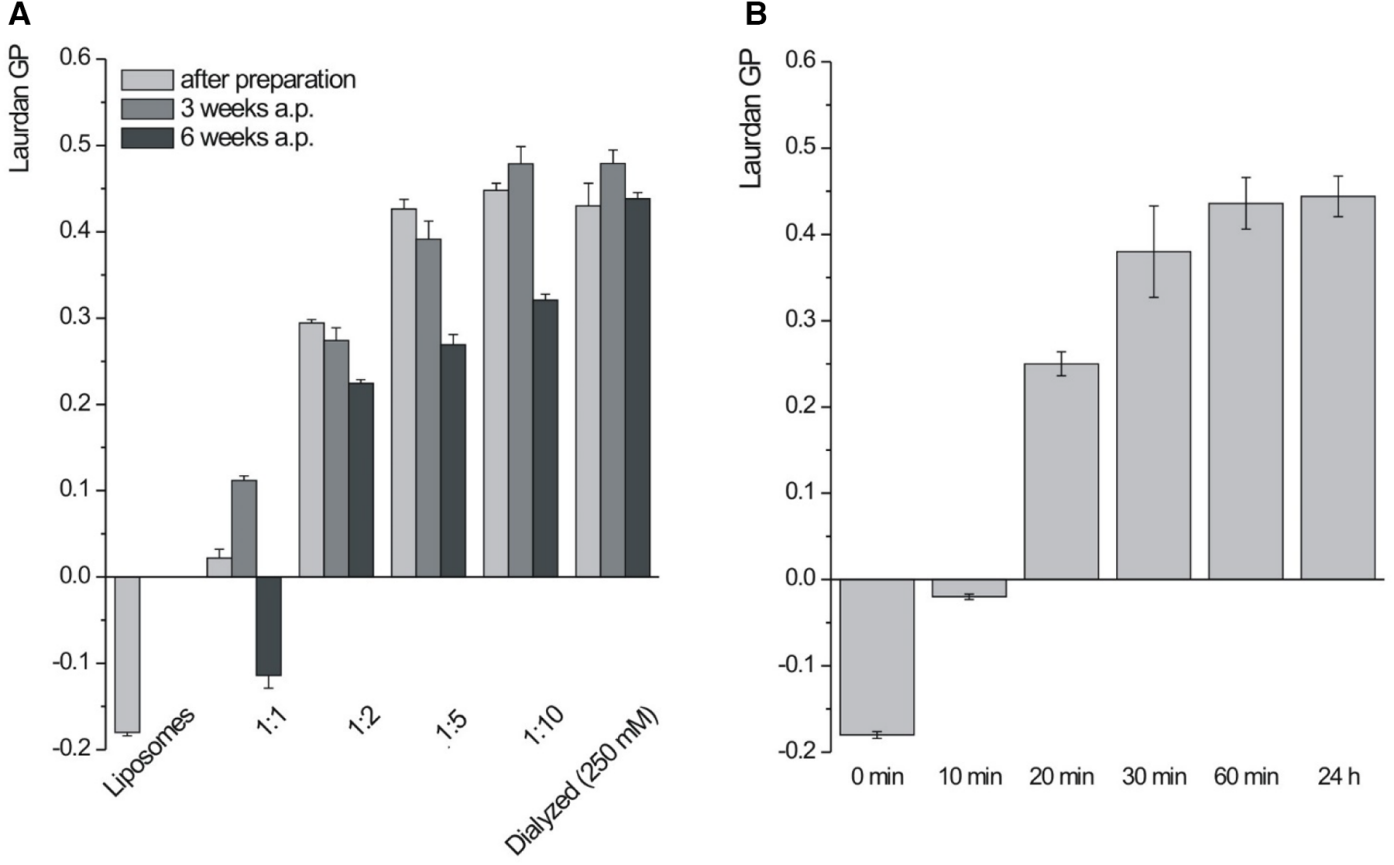
Laurdan GP (*n* = 3) of SPS-19 formulations measured after preparation and after storage (**A**) and during dialysis (**B**) cochleate suspensions were prepared by controlled mixing (80 µl/s) or dialysis. 25 mM EDTA was added to prepare liposomes and the calcium chloride concentration was adjusted accordingly to reach the desired lipid/calcium ion molar ration between 1:1 and 1:10.

**Figure 7:**
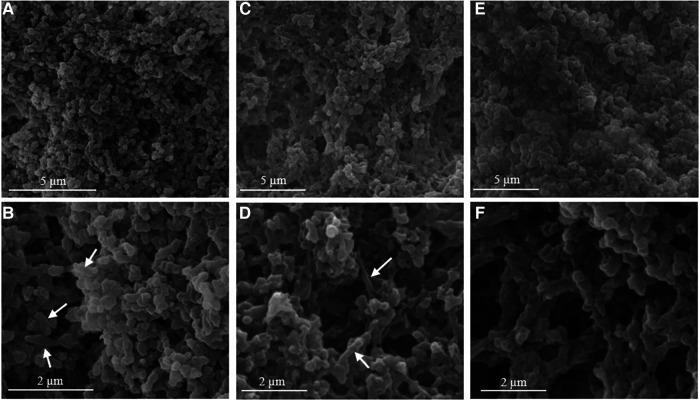
Representative SEM images of SPS-10 (**A,B**) and SPS-19 cochleate suspensions prepared by dialysis (**C,D**) and by controlled mixing (80 µl/s, lipid/calcium ion molar ratio of 1:5, **E,F**). Note the cylindrical cochleate particles marked with an arrow.

Independently on the lipid/calcium ion ratio, flocculated suspensions were obtained and the characteristic SAXD reflection (d-spacing 4.9 nm) was detected in all suspensions shortly after preparation (not shown). As expected, the aggregation tendency (aggregate size) increased with increasing amounts of added calcium chloride. There was also an increase in GP with increasing lipid/calcium molar ratio (Figure 6A) up to a ratio of 1:5 reaching then GP values between 0.4 and 0.5 (Figure 6A). However, GP values declined distinctly during storage in all samples prepared by direct mixing (Figure 6A). It can be speculated that the EDTA in the suspension interferes with the particle structure over time. To circumvent this problem, cochleate suspensions were prepared by dialysis. The process of lipid dehydration during dialysis could clearly be followed by the increasing Laurdan GP values and a plateau was reached after about 1 h (Figure 6B). Most importantly, the GP values did not decrease distinctly upon storage (Figure 6A) indicating improved stability compared to the samples prepared by direct mixing. Remarkably, the SPS-19 cochleate suspensions prepared with lipid/calcium chloride molar ratio of 1:5 and by dialysis had a rather similar morphology as those prepared from the purer SPS-10 (Figure 7). Importantly, some cochleates with the typical cylindrical particle shape could be detected (marked with arrows in Figures 7B,D).

## 4. Discussion

Overall, particles with a tightly packed and dehydrated lamellar structure could be prepared from soybean lecithin-derived phosphatidylserines. The formulation with a resemblance closest to the cylindrical DOPS cochleates was obtained from the soy-PS with highest purity (SPS-10, PS ≥ 96%). In both formulations, cochleate cylinders were co-existing with more compact particles. The similar GP-values and the presence of the characteristic sharp SAXD reflection indicate a highly ordered lipid structure. The shorter lamellar repeat distance (d-spacing, 4.9 nm) of the soy-PS particles is in good agreement with results obtained in a recent study (9) and can be explained by the mixed fatty acid chains in this naturally derived lipid. For dipalmitoyl phosphatidylserine (DPPS)-calcium, for example, the lamellar repeat distance is about 4.5 nm (26) and thus distinctly smaller than that of DOPS-calcium [5.1 nm (14)].

As the lecithin-derived lipids may contain residual calcium ions from the processing stage, addition of EDTA was needed to facilitate complete lipid dispersion in buffer and subsequent vesicle formation. However, there are some concerns with respect to an interference with the cochleate structure. Indeed, decreasing GP values measured during storage for the SPS-19 formulations with remaining EDTA has been observed in this study despite the rather large amount of added calcium ions. Sodium citrate with lower affinity towards calcium ions has been suggested to disperse cochleate aggregates (21) and may present an alternative to facilitate the dispersion of calcium-SPS. However, low water solubility of the formed calcium citrate is the limiting factor in this case. Preparation of cochleate suspension by dialysis, where the EDTA is removed simultaneously to the addition of calcium, presents an elegant alternative as stable suspensions were obtained this way.

Suspensions which were prepared from lipids with lower purity and lipid/calcium ion molar ratios of 1:1 (sodium salts) or 1:2 (calcium salts) resulted in particles with less well-ordered structures and dehydration (broader and less intensive SAXD reflection, lower GP values, no formation of cylindrical particles). However, the well-defined lamellar arrangement seen in electron microscopy and similar d-spacing as determined for SPS-10 cochleates indicate that at least some regions in the particles had a lipid organization and headgroup dehydration similarly to particles obtained from the purer PS. The content of neutral lipids such as phosphatidylcholine (PC) is likely the determining factor for the overall less well-ordered particle structure, and one may expect at least a partial segregation of the lipids in the lamellar structure. Addition of calcium ions to vesicles composed of an equimolar mixture of DOPS and DOPC, for example, resulted in segregation of DOPS in the tightly packed lipid organization of cochleates, while DOPC remained in fluid-phase (27). Considering the lipids used in the present study, the PC content specified by the manufacturer ranged from < 1% in the purified phosphatidylserine to above 5% but might be higher in the lipids with no specified PC content. Therefore, PC may introduce less ordered domains in the lipid particles resulting in lower Laurdan GP values and a broader SAXD reflections. For the investigated SPS-calcium salt (SPS-19), increasing amounts of calcium ions resulted in a higher degree of dehydration (higher Laurdan GP value). A similar effect has been described for mixtures of phosphatidic acid and phosphatidylcholine (28).

In conclusion, cochleate particles with similar structure and thus functionality as DOPS cochleates can be prepared from lecithin-derived phosphatidylserine with high head group purity (>96%) despite some differences in particle morphology (aspect ratio). Particles with a well-organized lamellar dehydrated structure could also be obtained from less pure SPS lipids, however, with a lower degree of structural order and dehydration depending on the lipid/calcium ion ratio. Importantly, the results of the study indicate the feasibility of direct processing of a calcium SPS salt by adding EDTA for liposome formation followed by dialysis to remove EDTA and add calcium ions for the formation of cochleates. Considering drug incorporation, small domains with less ordered structure may even be advantageous, as the loading capacity in the highly ordered, rigid lipid structure of cochleates can be expected to be limited for most drugs. Altogether, naturally derived phosphatidylserines present an interesting and promising option for further development of cochleate formulations for drug delivery.

## Data Availability

The original contributions presented in the study are included in the article/**Supplementary Material**, further inquiries can be directed to the corresponding author.
