# Supplementary material for: Feasibility of the preparation of cochleate suspensions from naturally derived phosphatidylserines
Source: Front Med Technol. 2023 Sep 6;5:1241368. doi: 10.3389/fmedt.2023.1241368 (PMC10512065; doi:10.3389/fmedt.2023.1241368)

Supplementary Material

Feasibility of the Preparation of Cochleate Suspensions from Naturally Derived Phosphatidylserines

Søren Kristensen, Khadeija Hassan, Nadia Skarnager Andersen, Frank Steiniger and Judith Kuntsche*

*** Correspondence:** Judith Kuntsche: kuntsche@sdu.dk

<u>Content:</u>

Supplementary Figure S1. Additional high-resolution SEM (a-d) and cryo-SEM (e-h) images of DOPS cochleates,

Supplementary Figure S2. Additional high-resolution SEM images of SPS-10 cochleates.

Supplementary Figure S3. Comparison of DOPS and SPS-10 cochleates prepared by the trapping method and by controlled mixing (80 µl/s). SEM images (a), Laurdan GP (b) and SAXD (c).

Supplementary Figure S4. SEM images of suspensions prepared from SPS with different purities: SPS-10 without (a) and with addition of 10 mM EDTA (b), SPS-13 (c), SPS-14 (d), SPS-15 (e) and SPS-16 (f).

Supplementary Figure S5. Determination of the lamellar thickness from the TEM images. 100 x 100 nm region of interest (ROI, marked with yellow box) for analysis of the bilayer spacing using the Fast Fourier Transform (FFT) function in ImageJ 1.52p (http://imagej.nih.gov/ij). Repeat distances are presented as mean ± SD (n = 4).

Supplementary Figure S1. Additional high-resolution SEM (a-d) and cryo-SEM (e-h) images of DOPS cochleates.

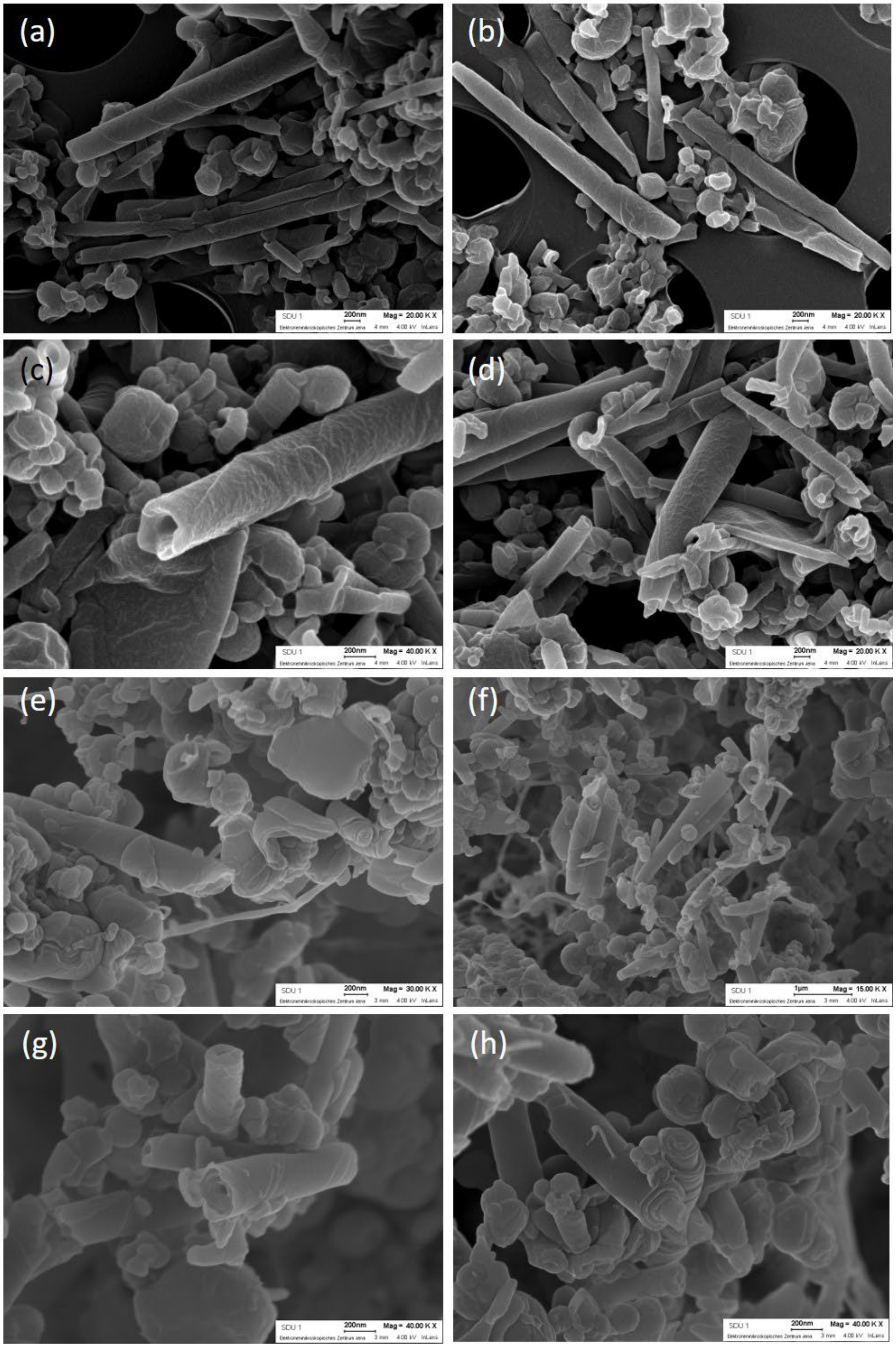

Supplementary Figure S2. Additional high-resolution SEM images of SPS-10 cochleates.

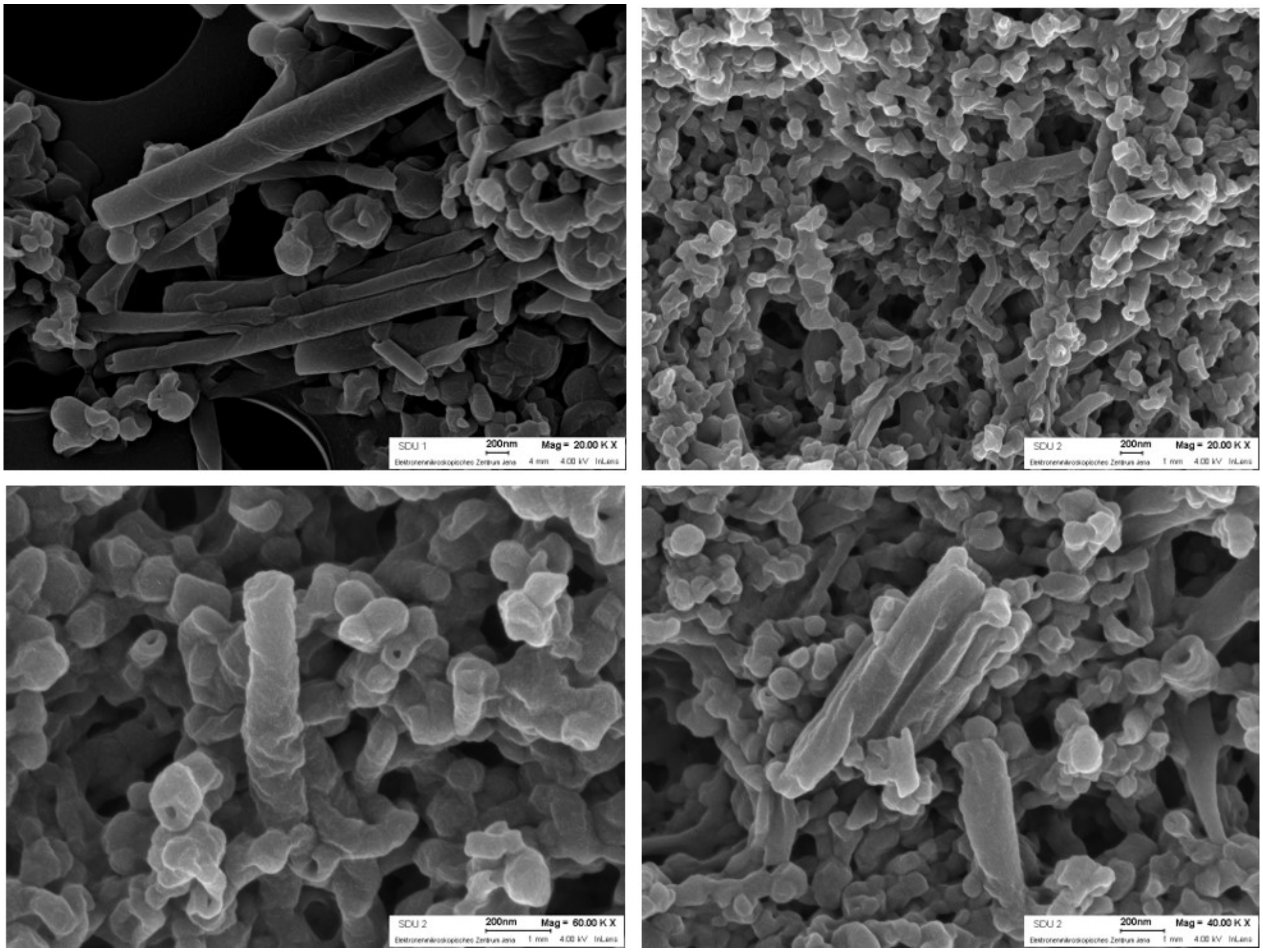

Supplementary Figure S3. Comparison of DOPS and SPS-10 cochleates prepared by the trapping method and by controlled mixing (80 µl/s): SEM images (a), Laurdan GP (b) and SAXD (c).

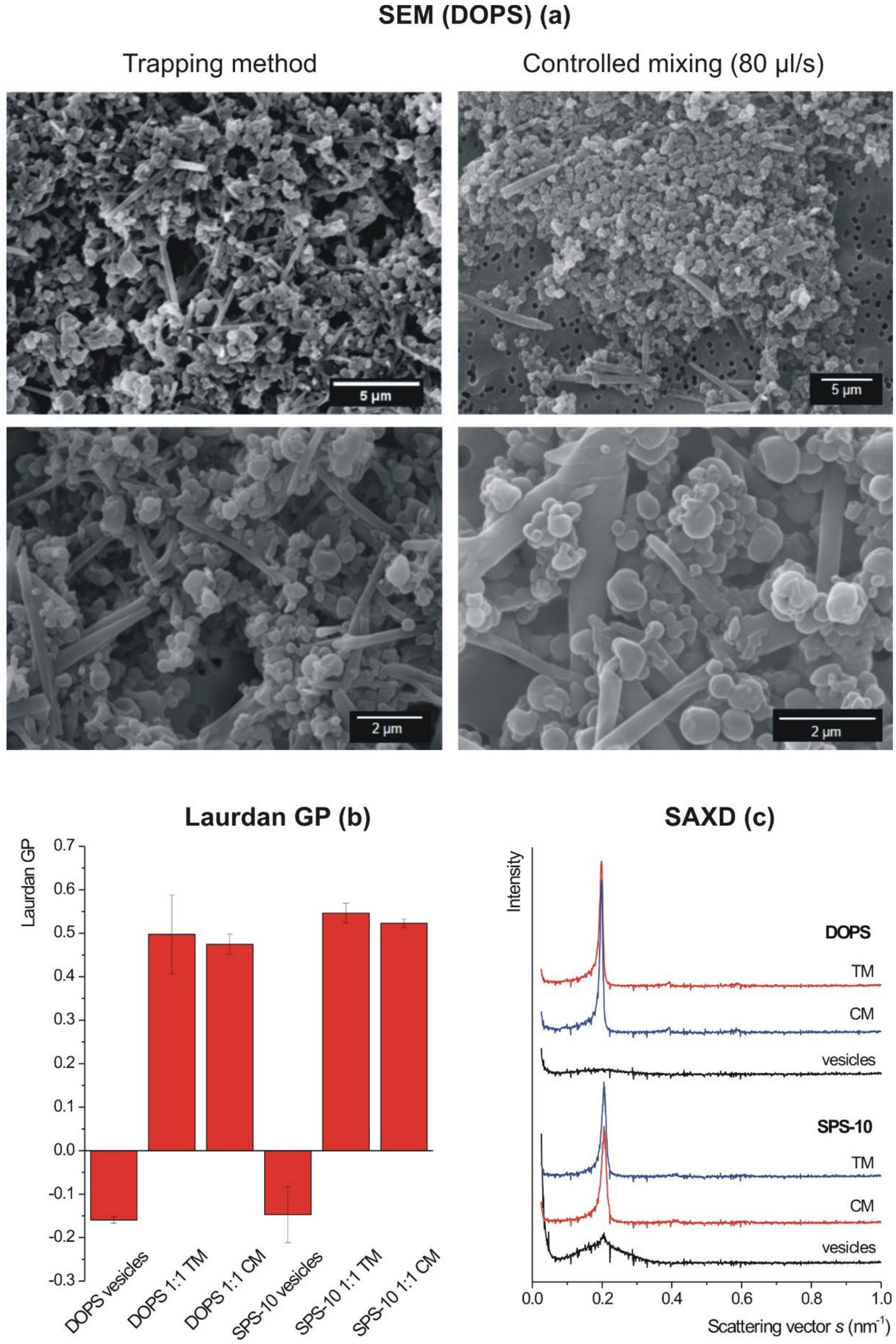

Supplementary Figure S4. SEM images of suspensions prepared from SPS with different purities: SPS-10 without (a) and with addition of 10 mM EDTA (b), SPS-13 (c), SPS-14 (d), SPS-15 (e) and SPS-16 (f).

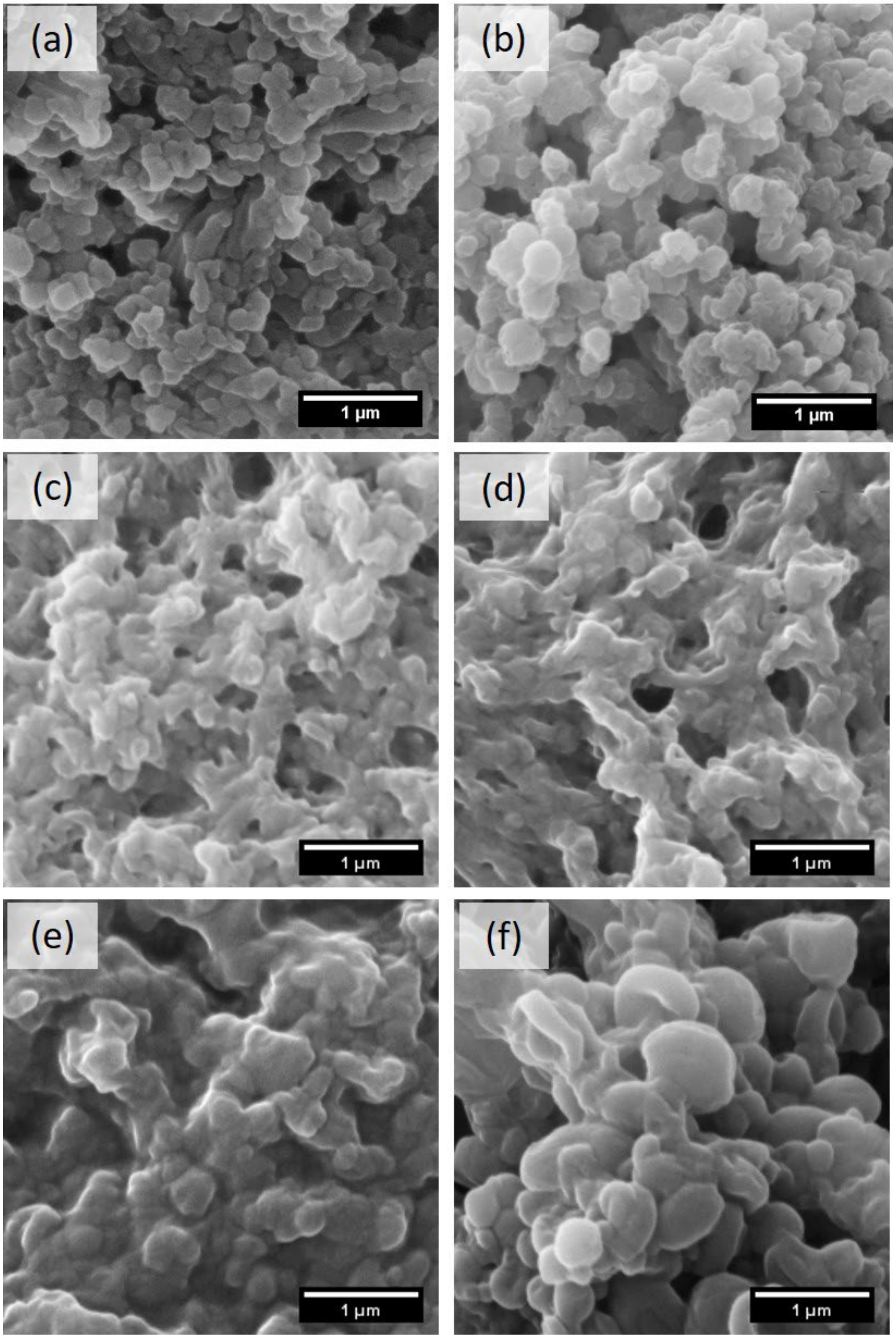

Supplementary Figure S5. Determination of the lamellar thickness from the TEM images. 100 x 100 nm region of interest (ROI, marked with yellow box) for analysis of the bilayer spacing using the Fast Fourier Transform (FFT) function in ImageJ 1.52p (http://imagej.nih.gov/ij). Repeat distances are presented as mean ± SD (n = 4).

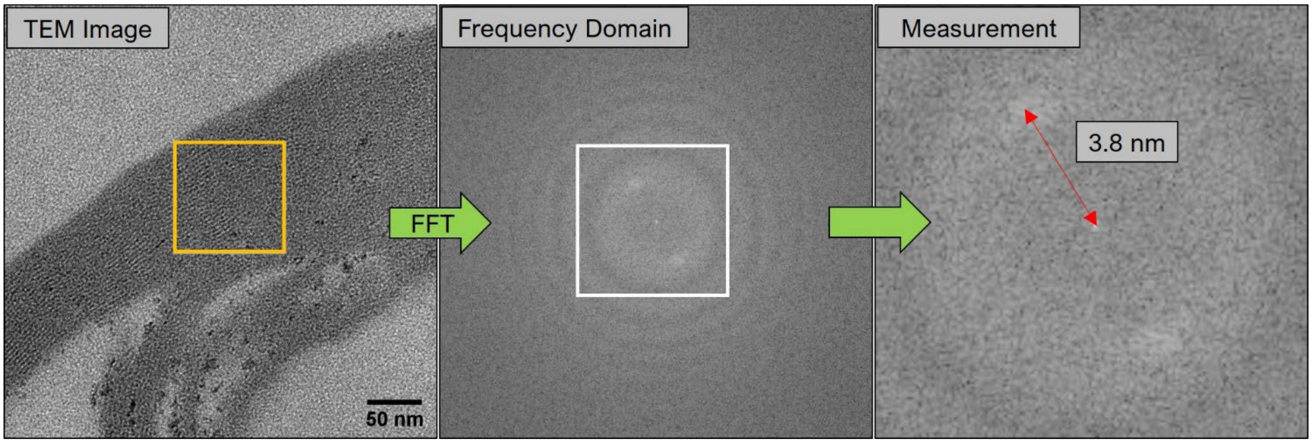

Supplement: Supplementary file 1 [file Datasheet1.pdf]
